# Risk factors for postoperative opioid prescription after benign hysterectomy

**DOI:** 10.1016/j.eurox.2026.100471

**Published:** 2026-06-23

**Authors:** Sophia Ehrström, Jan Baekelandt, Karin Källén, Andrea Stuart

**Affiliations:** a Karolinska Institutet, Department of Clinical Sciences, Division of Obstetrics and Gynaecology, Danderyd Hospital, Stockholm, Sweden; b Department of Gynecology, Imelda Hospital, Bonheiden, Belgium; c Centre for Reproductive Epidemiology, Lund University, Sweden; d Institution of Clinical Sciences, Department of Obstetrics and Gynecology, Lund University, Sweden

**Keywords:** Hysterectomy, Benign, Risk factors for opioid use, Opioid crisis, National data, Quality register

## Abstract

**Objective:**

To identify risk factors for dispensed opioid prescriptions after benign hysterectomy.

**Design:**

A nationwide register-based cohort study including 50,601 hysterectomies with complete data from the Swedish National Quality Register for Gynecological Surgery (GynOp). Data were linked to the Swedish National Drug Register, the National Patient Register, and Statistics Sweden. Logistic regression analyses were performed in three steps: adjusting for demographics, intraoperative factors, and postoperative complications.

**Results:**

Increasing age was associated with a lower risk of postoperative opioid dispensing (aRR 0.56 for >70 vs <50 years; 95% CI 0.49–0.64). Lower risk was also observed for hysterectomy due to prolapse (aRR 0.63; 95% CI 0.56–0.72) and cervical dysplasia (aRR 0.86; 95% CI 0.77–0.95). Modestly increased risks were found among patients using antihypertensive or cardiac medications (aRR 1.13; 95% CI 1.05–1.21), with previous cesarean section (aRR 1.10; 95% CI 1.05–1.16), BMI 30–39 (aRR 1.12; 95% CI 1.02–1.24), smoking (aRR 1.12; 95% CI 1.02–1.22), previous abdominal surgery (aRR 1.22; 95% CI 1.16–1.29), or previous vaginal surgery (aRR 1.15; 95% CI 1.06–1.24).

Substantially increased risks were associated with preoperative psycholeptics (aRR 1.65; 95% CI 1.55–1.76), antidepressants (aRR 1.22; 95% CI 1.13–1.32), immunomodulators (aRR 1.45; 95% CI 1.11–1.90), endometriosis (aRR 1.58; 95% CI 1.36–1.83), open surgery (aRR 1.43; 95% CI 1.33–1.54), and postoperative complications (aRR 1.46; 95% CI 1.40–1.53). The strongest predictor was region: surgery in Stockholm was associated with more than double the risk (aRR 2.52).

**Conclusions:**

Postoperative opioid dispensing after benign hysterectomy is primarily driven by patient-related factors and regional prescribing practices. Despite increasing adoption of minimally invasive techniques, opioid dispensing rose by 8% annually from 2005 to 2022, underscoring the need for targeted strategies to reduce unnecessary postoperative opioid exposure.

## Introduction

Hysterectomy is one of the most performed surgeries in women in the United States and in Sweden. There has been an ongoing opioid crisis in the US [1] and worldwide since almost a decade. In 2023, over 80 000 opioid overdose deaths were estimated in the USA. The risk of persistent opioid use after hysterectomy has been estimated to 5–6% in the US, and in 7.1% in Sweden [2], [3].

Previous studies have identified associations between surgical procedures and new persistent use of opioids [4] and have also tried to define optimal length of opioid pain medication prescription after common surgical procedures [5]. Studies from major surgery have identified patient factors [6] that could influence opioid consumption postoperatively, and the use of an opioid free ERAS-concept has shown a significant reduction of the use of opioids postoperatively [7]. Opioid consumption after benign hysterectomy has been studied in meta-analyses and smaller randomized controlled studies [8], [9]. Intraoperative factors such as endometriosis, adhesions and smaller uteri have been suggested as risk factors for a higher opioid consumption postoperatively [10]. Peri- and postoperative complications are well known factors that could raise the opioid consumption postoperatively [11], [12], [13], [14].

Minimal invasive surgery reduces postoperative complications and pain, compared to laparotomy [11], [12], [13], [14]. However, the prescription of opioids has increased during a time where minimal invasive surgery has become the new standard of care for hysterectomy in the US, and in Sweden [15], [2], [3]. The aim of our study was to study pre- and perioperative factors that influence the risk of dispensed postoperative opioid prescriptions.

## Material and methods

Data was merged from four Swedish National based registries from a cohort of 50601 hysterectomies for benign disease, 2005–2022.

Women who have had a hysterectomy, with or without concomitant adnexal surgery were identified from The Swedish National Quality Register for Gynecological Surgery (GynOp). [16].

The personal identification number was used to retrieve data from three national Swedish registries; Swedish Prescribed Drug Register (PDR), Statistics Sweden and the Swedish National Patient Register (NPR).

### Description of registries

GynOp contains preoperative, intraoperative, and postoperative information regarding gynecological surgery since 1997. The data registration rate is high, and the validated register provides complementary data with high accuracy [17], [18].

Data is collected from both the surgeon and the patient. The patient fills in a preoperative questionnaire to collect baseline demographic data and fills in a postoperative questionnaire eight weeks and 12 months after surgery regarding patient-reported complications. The surgeon confirms or rejects the patient-reported complications in the Register.

Demographic variables are filled in at the time of surgery (age, body mass index (BMI), tobacco use, previous abdominal surgery, and previous cesarean section), type of surgery (hysterectomy and/or adnexal surgery), primary incision (abdominal, laparoscopic, robotic, or vaginal), conversion to laparoscopy/laparotomy and indication for surgery. Postoperatively, the surgeon registers surgical data such as uterus weight, blood loss, and duration of surgery. Definition of complication: A tick box description of perioperative complications found in Gyn OP Register was used and also from the Patient Register described below.

### The Swedish National patient register

The Swedish National Patient Register contains data on patients admitted to all Swedish hospitals since 1987; date of admission, date of discharge and diagnoses and procedural codes registered for each admission. Codes are classified according to the International Classification of Diseases, 10th Revision codes. Indications for hysterectomy were defined as; endometriosis N80, prolapse N81, dysmenorrhea N94, cervical dysplasia N87, endometrial hyperplasia N85, menometrorrhagia N92, postmenopausal bleeding N95, and myoma D25. Indication for the hysterectomy was found from both the GynOp and the Patient Register.

Intraoperative complications were defined as; urinary bladder injury (ICD code S37.2) (procedural code suture of urinary bladder KCH 00, KCH96), ureter injury (ICD code: S37.1)(procedural code suture of ureter: KBH00, KBH96), bowel injury (ICD code S36.4–66) (procedural code suture of rectum JGA60), accidental puncture and laceration during a procedure, not elsewhere classified (T81.2) or vascular complications following a procedure, not elsewhere classified (T81.7). ICD and procedural codes were identified from the Patient Register. A tick box description of perioperative complications found in Gyn OP Register was also used and merged with the data from the Patient Register.

### Swedish drug register

The Drug Register was established in 2005 and is maintained by the Swedish National Board of Health and Welfare. It contains data on all drugs dispensed at pharmacies in Sweden. Pharmaceutical consumption is classified according to the Anatomic Therapeutic Chemical Classification (ATC). Dispensed prescriptions of drugs were extracted to identify preexisting comorbidities such as cardiovascular disease or diabetes (3 months prior to surgery) and to assess postoperatively dispensed opioid prescriptions (ATC code N02A) (1–21 days after surgery). The time period of 21 days was chosen to include postoperative prescriptions with a connection to the surgery and restricted to 21 days after surgery to minimize the effect of other future events that could influence prescriptions of opioids. The register provides no information regarding opioid dosage, duration of treatment or quantity prescribed.

Data regarding drug consumption 3 months prior to surgery was extracted from the Drug Register; A10A insulin, A10B oral antidiabetic agents, B01 antithrombotic agents, C01 cardiac therapy, C02 antihypertensives C03 diuretics, C04 peripheral vasodilators, C05 vasoprotectives, C07 beta blocking agents, C08 calcium channel blockers, C09 agents acting on the renin-angiotensin system, C10 lipid modifying agents, N05 psycholeptics, N06A antidepressants, N06B psychostimulants, H02AB glucocorticosteroids, L01XC monoclonal antibodies, L03 immunostimulating drugs, L04 immunosuppressing drugs.

### Statistics Sweden

Statistics Sweden is a government agency that produces official statistics. Data regarding country of birth was obtained from Statistics Sweden.

### Statistical methods

Crude and adjusted risk ratios (RR, aRR) for postoperatively dispensed opioid prescriptions were estimated using modified Poisson regression with robust error variance. Covariates included age, BMI, smoking, reproductive history, previous abdominal surgery, country of birth, education, indication for hysterectomy, surgical approach, uterine weight, operative duration, blood loss, conversion to laparotomy, perioperative complications, postoperative complications, and preoperative medications (diabetic, cardiac/antihypertensive, psycholeptics/psychostimulants, antidepressants, immunoregulation drugs, or none).

Three sequential models were fit: [1] demographics, [2] demographics plus operative factors, and [3] fully adjusted including postoperative complications. Results are reported as RR/aRR with 95% confidence intervals, with statistical significance defined as P < 0.05. Analyses were restricted to complete cases.

All analyses were performed using IBM SPSS Statistics, Version 28.0 (IBM Corporation, Armonk, NY, USA).

Ethical permission with number 2022–01489–01 was obtained from the Swedish National Ethical Board.

## Results

Data regarding 50 601 hysterectomies performed 2005–2022 was extracted from the Swedish GynOp Register. Records with complete data were included for analysis. Table 1 shows descriptive data and the rate of a postoperatively dispensed prescription of opioids.

**Table 1:** Rate of postoperative opioid prescription by surgical and patient characteristics.

**Characteristic**	**Postoperative opioids, n (%)**	**No postoperative opioids, n (%)**
**Total**	8594 (17.0)	42007 (83.0)
**Mode of hysterectomy**		
Abdominal	4450 (17.8)	20572 (82.2)
Laparoscopic	1307 (18.2)	5880 (81.8)
Robotic	1092 (19.8)	4418 (80.2)
Vaginal	1745 (13.5)	11137 (86.5)
**Uterine weight (g)**		
≤ 300	5432 (16.6)	27280 (83.4)
301–500	1230 (17.3)	5898 (82.7)
501–1000	1335 (17.7)	6208 (82.3)
> 1000	597 (18.6)	2621 (81.4)
**Surgical duration**		
	768 (22.5)	2645 (77.5)
45–59 min	842 (14.5)	4977 (85.5)
60–89 min	2420 (15.3)	13425 (84.7)
90–120 min	2103 (16.7)	10502 (83.3)
≥ 120 min	2461 (19.1)	10456 (80.9)
**Perioperative complications**	415 (22.5)	1431 (77.5)
**Blood loss (ml)**		
	7530 (16.5)	37972 (83.5)
500–999	810 (20.1)	3210 (79.9)
≥ 1000	191 (23.8)	611 (76.2)
**Conversion to laparotomy**	236 (24.0)	749 (76.0)
**Previous abdominal surgery**	1749 (21.5)	6375 (78.5)
**Reproductive history**		
Nulliparous	946 (20.0)	3793 (80.0)
Parous, no CS	5773 (15.5)	31393 (84.5)
1 previous CS	1159 (20.7)	4435 (79.3)
≥ 2 previous CS	716 (23.1)	2386 (76.9)
**BMI (kg/m²)**		
	77 (18.6)	337 (81.4)
18.5–24.9	3457 (16.1)	17968 (83.9)
25–29.9	2985 (16.7)	14902 (83.3)
30–34.9	1469 (18.8)	6362 (81.2)
35–39.9	456 (19.8)	1845 (80.2)
≥ 40	150 (20.2)	593 (79.8)
**Age (years)**		
	5713 (18.8)	24739 (81.2)
50–59	2088 (16.7)	10408 (83.3)
60–69	509 (11.5)	3922 (88.5)
≥ 70	284 (8.8)	2938 (91.2)
**Smoking**	505 (19.9)	2034 (80.1)
**Preoperative medications**		
Diabetic drugs	98 (18.8)	422 (81.2)
Cardiac/hypertensive drugs	953 (19.1)	4037 (80.9)
Psycholeptics/stimulants	1235 (30.2)	2853 (69.8)
Antidepressants	765 (27.1)	2059 (72.9)
Immunoregulatory drugs	54 (27.7)	141 (72.3)
**Educational level**		
Comprehensive school	964 (15.9)	5087 (84.1)
Secondary upper	4555 (17.0)	22318 (83.0)
Post-secondary < 3 years	1039 (17.0)	5068 (83.0)
University	2036 (17.6)	9534 (82.4)
**Need for interpreter**	86 (16.4)	437 (83.6)
**Region in Sweden**		
Stockholm	1905 (42.1)	2624 (57.9)
Central	1336 (13.2)	8768 (86.8)
South-East	1026 (14.1)	6240 (85.9)
Southern	2250 (19.5)	9269 (80.5)
Western	1186 (10.9)	9708 (89.1)
Northern	891 (14.2)	5398 (85.8)
**Indication for surgery**		
Prolapse	357 (7.8)	4215 (92.2)
Endometriosis	195 (41.1)	279 (58.9)
Pain/pressure	1093 (20.6)	4218 (79.4)
Postmenopausal bleeding	194 (16.2)	1002 (83.8)
Cervical dysplasia	427 (14.9)	2445 (85.1)
Menorrhagia	3990 (17.2)	19250 (82.8)
Dysmenorrhea	512 (25.0)	1537 (75.0)
Ovarian mass	411 (16.4)	2094 (83.6)
Other	784 (14.9)	4480 (85.1)
Not specified	631 (20.2)	2487 (79.8)

**Abbreviations:** CS, caesarean section; BMI, body mass index.

Table 2 shows crude and adjusted risk ratios for a postoperatively dispensed prescription of opioids. In the final model, after correcting for demographic factors, surgical history, indication for the surgery, and peri- and postoperative complications, the risk of postoperatively prescribed and dispensed opioids was decreased with higher age at surgery (aRR=0.56 >70 years vs <50 years; 95% CI 0.49–0.64). Although crude rates were higher with larger uteri, adjusted analysis showed lower risk of dispensed opioids with uterus weight more than 1 kg, (aRR=0.83; CI 0.75–0.91; >1 kg vs <300 g).

**Table 2:** Crude and adjusted risk ratios for postoperative opioid prescription.

*Modified Poisson regression models*				
**Factor**	**Category**	**Crude RR (95% CI)**	Adjusted RR\* (95% CI)	**Fully adjusted RR† (95% CI)**
**Age (years)**		1.00	1.00	1.00
	50–59	0.89 (0.85–0.94)	0.90 (0.85–0.95)	0.91 (0.86–0.96)
	60–69	0.61 (0.56–0.67)	0.69 (0.62–0.76)	0.71 (0.64–0.78)
	≥ 70	0.47 (0.42–0.53)	0.54 (0.48–0.62)	0.56 (0.49–0.64)
**Preoperative medications**	Diabetic drugs	0.99 (0.81–1.21)	0.98 (0.80–1.20)	0.98 (0.80–1.20)
	Cardiac/hypertensive drugs	1.05 (0.98–1.13)	1.14 (1.06–1.22)	1.13 (1.05–1.21)
	Psycholeptics/stimulants	1.76 (1.65–1.88)	1.68 (1.57–1.79)	1.65 (1.55–1.76)
	Antidepressants	1.34 (1.24–1.45)	1.24 (1.15–1.34)	1.22 (1.13–1.32)
	Immunoregulatory drugs	1.58 (1.21–2.06)	1.47 (1.12–1.92)	1.45 (1.11–1.90)
	None	1.00	1.00	1.00
**Reproductive history**	Nulliparous	1.28 (1.20–1.37)	1.00 (0.93–1.07)	1.00 (0.93–1.07)
	Parous, no CS	1.00	1.00	1.00
	Parous, previous CS	1.39 (1.32–1.46)	1.13 (1.07–1.19)	1.10 (1.05–1.16)
**Previous abdominal surgery**	Yes	1.34 (1.27–1.41)	1.24 (1.18–1.31)	1.22 (1.16–1.29)
	No	1.00	1.00	1.00
**BMI (kg/m²)**		1.14 (0.91–1.43)	1.10 (0.87–1.38)	1.07 (0.86–1.35)
	18.5–24.9	1.00	1.00	1.00
	25–29.9	1.03 (0.98–1.09)	1.02 (0.97–1.08)	1.03 (0.98–1.08)
	30–34.9	1.16 (1.09–1.24)	1.11 (1.04–1.18)	1.09 (1.03–1.16)
	35–39.9	1.23 (1.11–1.35)	1.16 (1.05–1.28)	1.12 (1.02–1.24)
	≥ 40	1.25 (1.06–1.47)	1.11 (0.94–1.30)	1.03 (0.87–1.22)
**Smoking**	Yes	1.18 (1.08–1.29)	1.12 (1.02–1.22)	1.11 (1.02–1.22)
	No	1.00	1.00	1.00
**Country of birth**	Nordic	1.00	1.00	1.00
	Europe (non-Nordic)	0.99 (0.88–1.11)	1.01 (0.89–1.14)	1.01 (0.90–1.14)
	Non-European	1.28 (1.20–1.36)	1.05 (0.98–1.12)	1.04 (0.97–1.11)
**Educational level**	Primary school	0.90 (0.84–0.98)	1.02 (0.95–1.11)	1.03 (0.95–1.12)
	Upper secondary	0.96 (0.91–1.01)	1.00 (0.95–1.06)	1.01 (0.95–1.06)
	Post-secondary < 2 years	0.97 (0.90–1.04)	1.00 (0.92–1.07)	1.00 (0.93–1.08)
	University	1.00	1.00	1.00
**Healthcare region**	Stockholm	1.00	1.00	1.00
	Central	0.31 (0.29–0.34)	0.28 (0.26–0.31)	0.29 (0.26–0.31)
	South-East	0.34 (0.31–0.36)	0.29 (0.26–0.31)	0.28 (0.26–0.31)
	Southern	0.46 (0.44–0.49)	0.44 (0.41–0.46)	0.44 (0.41–0.47)
	Western	0.26 (0.24–0.28)	0.24 (0.22–0.26)	0.23 (0.21–0.25)
	Northern	0.34 (0.31–0.37)	0.29 (0.27–0.32)	0.29 (0.27–0.32)
**Mode of hysterectomy**	Laparoscopic	1.00	1.00	1.00
	Abdominal	0.98 (0.92–1.04)	1.46 (1.36–1.56)	1.43 (1.33–1.54)
	Robotic	1.09 (1.01–1.18)	1.02 (0.94–1.11)	1.02 (0.94–1.11)
	Vaginal	0.75 (0.69–0.80)	1.13 (1.05–1.22)	1.15 (1.06–1.24)
**Uterine weight (g)**	≤ 300	1.00	1.00	1.00
	301–500	1.04 (0.98–1.11)	0.90 (0.85–0.96)	0.90 (0.84–0.96)
	501–1000	1.07 (1.00–1.13)	0.88 (0.82–0.94)	0.87 (0.81–0.93)
	> 1000	1.12 (1.03–1.22)	0.85 (0.78–0.94)	0.83 (0.75–0.91)
**Indication for hysterectomy**	Menorrhagia/pain/dysmenorrhea	1.00	1.00	1.00
	Prolapse	0.43 (0.38–0.48)	0.63 (0.56–0.71)	0.63 (0.56–0.72)
	Endometriosis	2.25 (1.95–2.60)	1.68 (1.45–1.95)	1.58 (1.36–1.83)
	Postmenopausal bleeding	0.89 (0.77–1.02)	1.10 (0.94–1.28)	1.10 (0.94–1.28)
	Cervical dysplasia	0.81 (0.74–0.90)	0.84 (0.76–0.93)	0.86 (0.77–0.95)
	Other	0.92 (0.87–0.97)	0.98 (0.93–1.04)	0.98 (0.93–1.04)
**Surgical time**	per 30-min increase	1.04 (1.03–1.06)	—	1.01 (1.00–1.03)
**Year of surgery**	Per one-year increment	1.08		1.08
**Perioperative complications**	Yes	1.34 (1.21–1.48)	—	1.08 (0.97–1.20)
	No	1.00	—	1.00
**Blood loss (ml)**		1.00	—	1.00
	500–999	1.22 (1.13–1.31)	—	1.07 (0.99–1.15)
	≥ 1000	1.42 (1.26–1.61)	—	1.05 (0.92–1.21)
**Postoperative complications**	Yes	1.59 (1.52–1.67)	—	1.46 (1.40–1.53)
	No	1.00	—	1.00

\*Adjusted for demographics. †Fully adjusted for demographics, surgical factors, and complications.

Patients with BMI 30–39 (aRR=1.12; 95% CI 1.02–1.24) previous CS (aRR=1.10; 95% CI 1.05–1.16) and previous abdominal surgery (aRR=1.22; 95% CI 1.16–1.29) had increased risk of dispensed postoperative opioids.

Patients with preoperative use of antihypertensive or cardiac drugs (aRR=1.13; 95% CI 1.05–1.21), psycholeptics (aRR=1.65 95% CI 1.55–1.76), antidepressants (aRR=1.22; 95% CI 1.13–1.32) or immunoregulatory drugs (aRR=1.45; 95% CI 1.11–1.90) had a higher risk of postoperatively dispensed opioids.

Hysterectomy due to prolapse (aRR=0.63; 95% CI 0.56–0.72) and cervical dysplasia (aRR =0.86; 95% CI 0.77–0.95) had the lowest risk for postoperatively dispensed opioids compared to surgery performed due to menorrhagia, dysmenorrhea and pain.

Endometriosis as indication for surgery (aRR=1.58; 95% CI 1.36–1.83), open surgery (aRR= 1.43; 95% CI 1.33–1.54), and postoperative complications (aRR=1.46; 95% CI 1.40–1.53), increased the risk of a postoperatively dispensed opioid prescription substantially.

Being operated in Stockholm, the capital of Sweden, was the largest risk factor for a dispensed prescription of opioids, aRR: 2.52 (95% CI 2.37–2.67) in the fully adjusted compared to not being operated in Stockholm.

As shown in Table 2, an eight percent yearly increase in dispensed opioid prescription was evident 2005–2022. The adjusted models showed that the presence of perioperative events or complications had no impact on the 8 % yearly increase.

## Discussion

### Principal findings

In our study, we have identified several risk factors for a dispensed opioid prescription after benign hysterectomy. The main risk factors for a higher opioid use postoperatively were in fact patient related, such as taking antidepressants, immunomodulating or psychostimulating drugs preoperatively, and the risk did not increase if these patients had perioperative complications, though laparotomy, surgery for endometriosis and postoperative complications were also strong individual factors for postoperative opioid prescriptions.

The strongest risk factor for a dispensed postoperative opioid prescription was being operated in the Stockholm region, highlighting the importance of behavioral prescription patterns among gynecologists, based on local traditions rather than presence of perioperative or postoperative complications.

## Clinical implications

Opioid prescriptions may initiate the risk for addiction and associated health complications, which may lead to economic and social concerns for the individual, and for some overdose deaths. Drug overdose is the leading cause of unintentional injury-associated death in the United States. The opioid epidemic in the United States has had a profound impact, with approximately 47,000 deaths in 2018 and 2 million individuals diagnosed with opioid use disorder in 2017 [19], [20]. The financial burden is equally staggering, with an estimated economic cost of $1.021 trillion in 2017, comprising $471 billion attributable to opioid use disorder and $550 billion linked to fatal overdoses. The US Center for Disease Control and prevention (CDC) underscores the urgent need for comprehensive prevention, treatment, and policy interventions to mitigate opioid overuse devastating health and economic consequences. It is thus of great importance to identify risk factors for opioid use after hysterectomy [21], [22].

Our analysis presents previously known factors for higher opioid consumption after surgery, such as hysterectomy due to endometriosis [3], postoperative complications [22], and laparotomy [8], [13], [23], [24]. Patients with BMI 30–40 have a 10% increased risk [25].

Surgical factors such as bleeding, operation time, and perioperative complications had in fact a limited impact and uterine size > =300 g even lowered opioid prescriptions. Postoperative complications, surgery due to endometriosis or hysterectomy performed with laparotomy raised the risk of dispensed opioids. Laparotomy has declined the last decade. Minimal invasive surgical (MIS) techniques have well-known advantages such as decreased length of hospital stay, improved postoperative recovery, and decreased postoperative pain [11], [12], [13], [14]. The results clearly state that minimal invasive surgery should be chosen to reduce the need for opioid prescriptions after hysterectomy.

## Research implications

Interestingly, women using immunomodulating drugs have a higher risk of dispensing postoperative opioids. This patient group has not been suggested as a risk group in previous studies and we suggest a novel risk group for postoperatively dispensed opioid prescriptions. We hypothesize that women with a history of joint pain (rheumatic diseases) might have a higher risk for postoperative pain. Patients preoperatively using psycholeptics and antidepressants, showed 65% and 22% higher risk for postoperative opioid usage, indicating a vulnerability to pain, which is in line with other publications [4], [26]. We suggest that women using immunomodulating drugs, psycholeptics and antidepressants could possibly be targeted as patients in higher need of optimized analgesia during and after hysterectomy.

## Strengths and limitations

This study is based on a large, nationwide cohort of approximately 50000 hysterectomies with complete data, providing strong statistical power and generalizability in a European country. The use of multiple validated national registries ensured comprehensive coverage of demographic, surgical, and information of dispensed prescriptions. The prospective registry design with both surgeon-reported and patient-reported outcomes minimized recall bias. The sequential multivariable modeling allowed clear separation of demographic, perioperative, and postoperative risk factors.

The limitations are that prescription data reflect dispensed opioids, and not actual consumption. Information regarding indication-specific symptom burden is not available. In addition, patients admitted for longer hospital stays may receive opioids during admission but not require prescriptions at discharge, which could lead to an underestimation of postoperative opioid use in that specific group of patients. We have no data regarding opioid dosage and treatment length. Perioperative analgesic protocols and anesthesia type are important determinants of postoperative pain. Unfortunately, these variables are not available in the GynOp or linked registries and cannot be adjusted for.

One would expect a lower rate of dispensed prescriptions of opioids over time due to the increase of MIS, but we see quite the opposite development the last 20 years in Sweden, as studies from the US have also reported [15]. Our analyses show that dispensed prescriptions of opioids have in fact increased as much as 8% every year (260% cumulative effect over the 18-year period), despite the shift from laparotomy to dominating minimal invasive surgery in the US and in Sweden [16]. In a previous study, women undergoing vaginal hysterectomy were prescribed a significantly lower amounts of opioids than those undergoing laparoscopic or abdominal hysterectomies [15]. In another meta-analysis, hysterectomy route did not predict persistent opioid use postoperatively, whereas younger age, smoking, alcohol use, back pain, and fibromyalgia were associated with persistent opioid use [8].

Regardless of surgical technique, it is the region for the surgery that influences the risk of dispensed opioid prescription most. Since the largest risk factor of dispensed opioid prescriptions was undergoing surgery in Stockholm, local customs rather than perioperative conditions or events impact postoperative risk for dispensed opioids. This fact underscores the critical responsibility of gynecologists in prescribing opioids judiciously, as their prescribing practices can significantly influence the trajectory of the global opioid crisis by potentially reducing over-prescription and preventing misuse, addiction, and associated health complications.

There is an absence of specific Swedish national guidelines for opioid prescription after hysterectomy, which may lead to variability and potential over-prescription in some geographical areas. We identify the need for guidelines to reduce opioid prescriptions, especially in uncomplicated procedures that have an expected lower risk for postoperative pain.

Another potential way forward is implementing the postoperative ERAS-concept on a larger scale. The ERAS-concept minimal use of opioids is feasible in major gynecologic surgery, for example hysterectomy and should be considered [7]. Transabdominal percutaneous and paracervical blocks could be used as they may also reduce the need of opioids postoperatively [27].

## Conclusion

Our study shows that postoperatively dispensed opioid prescription risks are primarily influenced by patient and physician factors alongside surgical postoperative complications, emphasizing the need for healthcare providers to recognize patient vulnerability, and challenge local practices that may elevate the risk of opioid dependence, thereby promoting safer pain management strategies for women undergoing hysterectomy.

## CRediT authorship contribution statement

**Jan Baekelandt:** Writing – review & editing, Supervision, Methodology. **Karin Källén:** Software, Methodology, Investigation, Formal analysis, Data curation. **Sophia Ehrström:** Writing – original draft, Methodology, Investigation, Formal analysis, Conceptualization. **Andrea Stuart:** Writing – review & editing, Supervision, Methodology, Funding acquisition, Data curation, Conceptualization.

## Funding information

The study was supported by grants from the Stig and Ragna Gorthon Foundation. The funders had no role in study design, data analysis, data interpretation, or writing of the report.

## Declaration of Competing Interest

The authors declare the following financial interests/personal relationships which may be considered as potential competing interests: Andrea Stuart reports financial support was provided by Helsingborg hospital, Department of Obstetrics ang Gynaecology. If there are other authors, they declare that they have no known competing financial interests or personal relationships that could have appeared to influence the work reported in this paper
